# Patients with Old Age or Proximal Tumors Benefit from Metabolic Syndrome in Early Stage Gastric Cancer

**DOI:** 10.1371/journal.pone.0089965

**Published:** 2014-03-05

**Authors:** Xiao-li Wei, Miao-zhen Qiu, Huan-xin Lin, Ying Zhang, Jian-xin Liu, Hong-mei Yu, Wei-ping Liang, Ying Jin, Chao Ren, Ming-ming He, Wei-wei Chen, Hui-yan Luo, Zhi-qiang Wang, Dong-sheng Zhang, Feng-hua Wang, Yu-hong Li, Rui-hua Xu

**Affiliations:** 1 Department of Medical Oncology, Sun Yat-sen University Cancer Center, State Key Laboratory of Oncology in South China, Collaborative Innovation Center for Cancer Medicine, Guangzhou, China; 2 Department of Preventive Care, Sun Yat-sen University Cancer Center, State Key Laboratory of Oncology in South China, Collaborative Innovation Center for Cancer Medicine, Guangzhou, China; The University of Hong Kong, China

## Abstract

**Background:**

Metabolic syndrome and/or its components have been demonstrated to be risk factors for several cancers. They are also found to influence survival in breast, colon and prostate cancer, but the prognostic value of metabolic syndrome in gastric cancer has not been investigated.

**Methods:**

Clinical data and pre-treatment information of metabolic syndrome of 587 patients diagnosed with early stage gastric cancer were retrospectively collected. The associations of metabolic syndrome and/or its components with clinical characteristics and overall survival in early stage gastric cancer were analyzed.

**Results:**

Metabolic syndrome was identified to be associated with a higher tumor cell differentiation (P = 0.036). Metabolic syndrome was also demonstrated to be a significant and independent predictor for better survival in patients aged >50 years old (P = 0.009 in multivariate analysis) or patients with proximal gastric cancer (P = 0.047 in multivariate analysis). No association was found between single metabolic syndrome component and overall survival in early stage gastric cancer. In addition, patients with hypertension might have a trend of better survival through a good control of blood pressure (P = 0.052 in univariate analysis).

**Conclusions:**

Metabolic syndrome was associated with a better tumor cell differentiation in patients with early stage gastric cancer. Moreover, metabolic syndrome was a significant and independent predictor for better survival in patients with old age or proximal tumors.

## Introduction

Gastric cancer (GC) is the fourth most common cancer in men and fifth in women, about 10% of annual deaths from cancer are attributed to GC. In 2011, 464,000 men and 273,000 women died from GC according to estimates [1]. The mortality of GC has declined steadily worldwide, especially cancer in the fundus and pylorus [2]. Compared with developed countries, the incidence and mortality is higher in developing countries. Over 70% of new cases and deaths occur in developing countries, and the highest incidence rates are in Eastern Asia, Eastern Europe, and South America [1].

Metabolic syndrome (MetS) was first described as syndrome X in 1988, the basis of MetS was insulin resistance [3]. Till now, a commonly agreed-upon standard of the definition has not been reached yet. But MetS has been similarly defined to be composed of a group of risk factors for cardiovascular disease, which including increased blood pressure, plasma glucose, triglycerides, and body mass index (BMI)/waist circumference, and decreased high density lipoprotein as well [4]. Interestingly, there have been plenty of studies investigating the association between MetS and cancers. MetS and/or its components have been found to be possible risk factors of several cancers, including endometrial cancer [5], [6], ovarian cancer [7], colorectal cancer [6], [8], [9], breast cancer [6], [10], lymphoma, multiple myeloma [11], thyroid cancer [12], cervical cancer [13], liver cancer [6], [14], [15], skin cancer [16], biliary tract cancer [17], pancreatic cancer [6], [18], [19], bladder cancer [6], [20] oesophageal cancer and GC [21], [22].

There also have been some researches about the influence of MetS and/or its components on survival in cancers. MetS was identified to be a risk factor for cancer related mortality as a whole in South Korea and America [23], [24]. Concerning the impacts on certain cancers, patients with MetS were more inclined to suffer from cancer related death in prostate cancer [25], [26], and MetS was associated with poor prognosis in breast cancer as well [26], [27]. In colon cancer, elevated glucose or diabetes mellitus and elevated hypertension were found to be associated with worse survival, while dyslipidemia was exactly the opposite, especially in patients with early stage disease [28]. But the prognostic value of MetS as a whole in the survival of colon cancer patients was controversial [28], [29]. In GC, a few studies showed that BMI had no association with survival, while decreased high-density lipoprotein was a predictor of worse survival [30], [31], [32]. To date, as far as we are concerned, no study concerning about the prognostic value of MetS in the survival of GC patients has been carried out. Thus we collected clinical and survival data of patients with early stage GC, who were diagnosed and received treatment in our hospital, to analyze whether pre-treatment status of MetS and/or its components have any impact on the overall survival (OS) in Chinese patients with early stage GC.

## Materials and Methods

### Ethics statement

All patients provided written informed consent for their information to be stored and used in the hospital database. Study approval was obtained from independent ethics committees at Cancer Center of Sun Yat-Sen University. The study was undertaken in accordance with the ethical standards of the World Medical Association Declaration of Helsinki.

### Study population

Based on the discharge diagnosis, 587 patients who were diagnosed with early stage GC and received treatment at Sun Yat-sen University Cancer Center in Guangzhou, China from March 23, 1999 to December 7, 2012 were included in this research. All patients were staged from I–III by the American Joint Committee on Cancer (AJCC) gastric cancer (GC) tumor-node-metastasis (TNM) stage system. In addition, all patients received operation for GC. The age range of patients included in our study was 24–83 years old. Cases without complete information about pre-treatment metabolic syndrome (MetS) were excluded. The data of pre-treatment MetS status and clinical information were retrospectively collected. For survival data, patients were followed-up by the follow-up department or at the outpatient department after discharge from hospital.

### Criteria for the definition of MetS adopted in our study

Through integrated consideration of different versions of the definition of MetS [4] and the availability of items in those versions of definition from our data, we defined MetS in this research according to the National Cholesterol Education Program's Adult Treatment Panel III (ATP III) (≧3 of 5 criteria necessary): 1). Impaired glucose regulation or diabetes mellitus, Fasting plasma ≧110 mg/dl (6.1 mmol/L). 2). Abdominal obesity, because we didn't find the records of abdomen circumference in clinical data, body mass index (BMI) ≧25 Kg/m^2^ was applied to be the substitute. 3). Triglycerides ≧150 mg/dl (1.7 mmol/L). 4). High density lipoprotein (HDL) ≦40 mg/dl (1.04 mmol/L) for male, ≦50 mg/dl (1.3 mmol/L) for female. 5). Hypertension, Systolic blood pressure ≧130/diastolic blood pressure ≧80 mm Hg. The data about each component of MetS was collected from clinical records before treatment.

### Statistical analysis

The statistical analyses were performed with SPSS for Windows V.13.0. A two tailed p value<0.05 was considered statistically significant. Differences of baseline clinical parameters between MetS positive and negative group were evaluated by chi-square test or Kruskal-Walli H test based on the type of the data and comparisons. Overall survival (OS) was the time interval from the date of diagnosis to death from GC, for patients who remained alive, the data were censored at the date of the last contact. OS curves were plotted with the Kaplan-Meier method, and differences were compared with log-rank test. Variables significantly prognostic in the univariate analysis were included in the multivariate survival analysis. Hazard ratio (HR) and 95% confidence interval (95% CI) were computed with the Cox proportional-hazards model.

## Results

### The association between MetS and baseline clinical characteristics

There were 587 cases analyzed, detailed information were listed in table 1. Among them, 79 cases (13.46%) were identified to meet the criteria of MetS, the rest of 508 cases (86.54%) were classified to be without MetS. Baseline clinical characteristics were compared between patients with and without MetS. As showed in table 1, there were no significant differences between patients with MetS and without MetS in gender, age, T stage (AJCC, 7th), N stage (AJCC, 7th), tumor size, tumor location, type of operation. While compared with patients with MetS, patients without MetS were testified to have a significantly more aggressive tumor cell differentiation (P = 0.036).

**Table 1:** Comparison of baseline clinical characteristics According to Metabolic Syndrome Status.

	No. of Patients (%)		
Characteristic	With MetS	Without MetS	P value
No. of patients	79(13.46)	508(86.54)	
Gender			0.082
Male	48(60.76)	358(70.47)	
Female	31(39.24)	150(29.53)	
Age (yr)			0.182
≧50	64(81.01)	376(74.02)	
<50	15(18.99)	132(25.98)	
T stage(AJCC, 7th)			0.605
T1a+T1b	11(13.92)	43(8.46)	
T2	7(8.86)	82(16.14)	
T3	1(1.27)	23(4.53)	
T4a+T4b	60(75.95)	360(70.87)	
N stage(AJCC, 7th)			0.205
N0	39(49.37)	177(34.84)	
N1	6(7.59)	84(16.54)	
T2	12(15.19)	118(23.23)	
N3a+N3b	22(27.85)	129(25.39)	
Tumor size (cm)			0.533
≤5	54(68.35)	329(65.31)	
>5	25(31.65)	179(34.69)	
Tumor location			0.111
Proximal*	52(65.82)	286(56.30)	
Distal*	27(34.18)	222(43.70)	
Degree of differentiation			**0.036**
Poorly or not differentiated, mucinous or signet ring adenocarcinoma	53(67.09)	390(76.77)	
Moderately differentiated addenocarcinoma	18(22.78)	102(20.08)	
Well differentiated addenocarcinoma	8(10.13)	16(3.15)	
Type of operation			0.083
Radical	71(89.87)	481(94.69)	
Palliative	8(10.13)	27(5.31)	

Abbreviations and explanations: AJCC: American Joint Committee on Cancer, MetS: metabolic syndrome, Proximal*: the fundus, cardia and body of stomach, Distal*: the pylorus and antrum of stomach.

### The impacts of Mets and/or its components on overall survival

**Figure 1:**
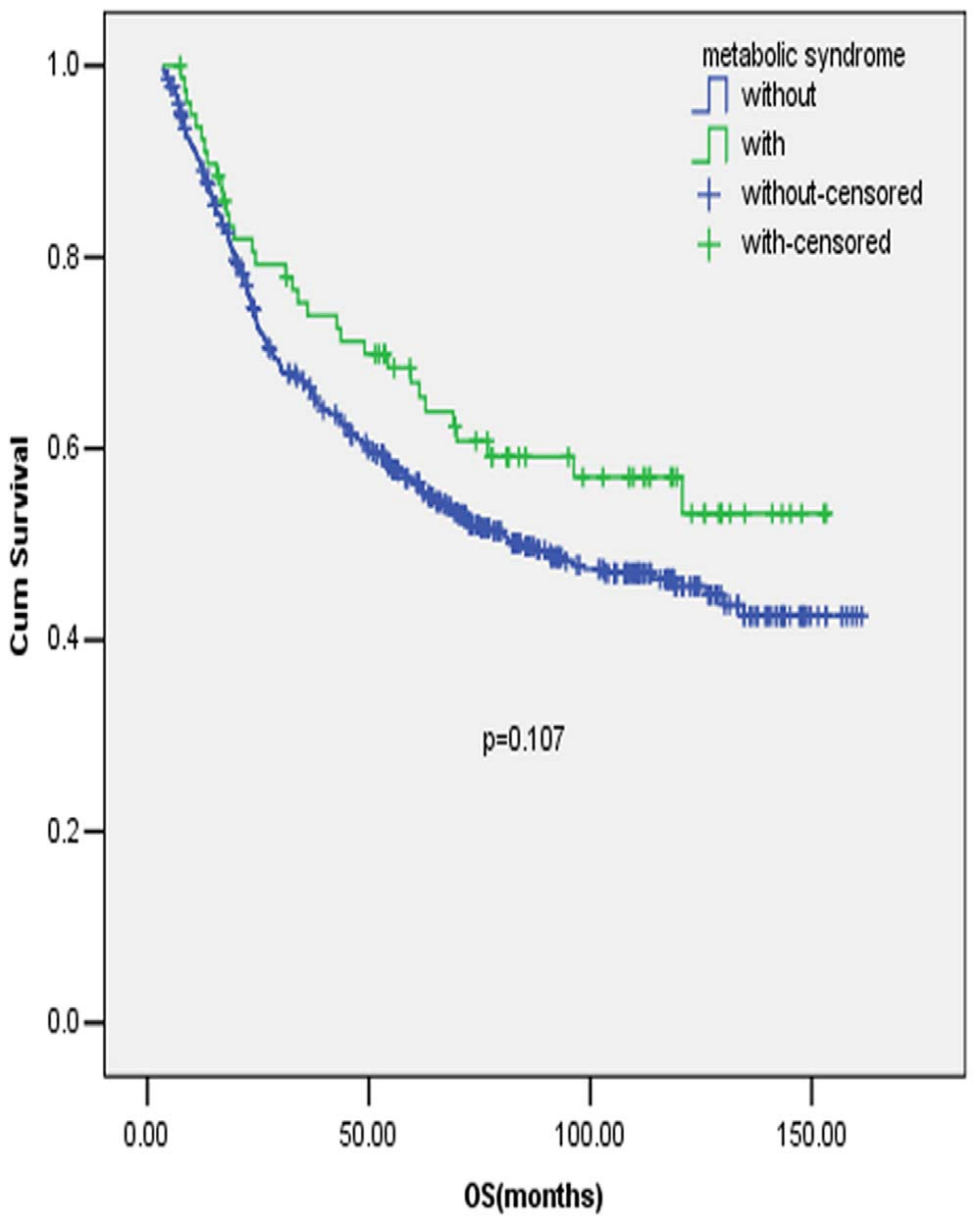
Prognostic value of MetS in early stage gastric cancer patients. Full legend: Patients with MetS had a trend of better survival compared with those without MetS in early stage gastric cancer (GC), but the difference was not statistically significant.

The survival analyses included 586 qualified cases with available survival information, and 1 case was excluded because of losing contact after discharge from our hospital. Judging from the survival curve by Kaplan-Meier method, there was a trend of better OS for patients with MetS compared with those without MetS (figure 1), but the difference was not statistically significant (P = 0.107). No apparent influence of the components of MetS on OS was found, either, nor did the number of items meeting the criteria of the definition of MetS have any important role in the OS of early stage GC.

Because previous studies found that MetS and/or its components were associated with higher risk of cardiac rather than non-cardiac GC, in addition, the association was not found in early adulthood [21], [22], [33], we further performed the survival analysis stratified by age (<50 y/≧50 y) and tumor location (Proximal/Distal).

**Figure 2:**
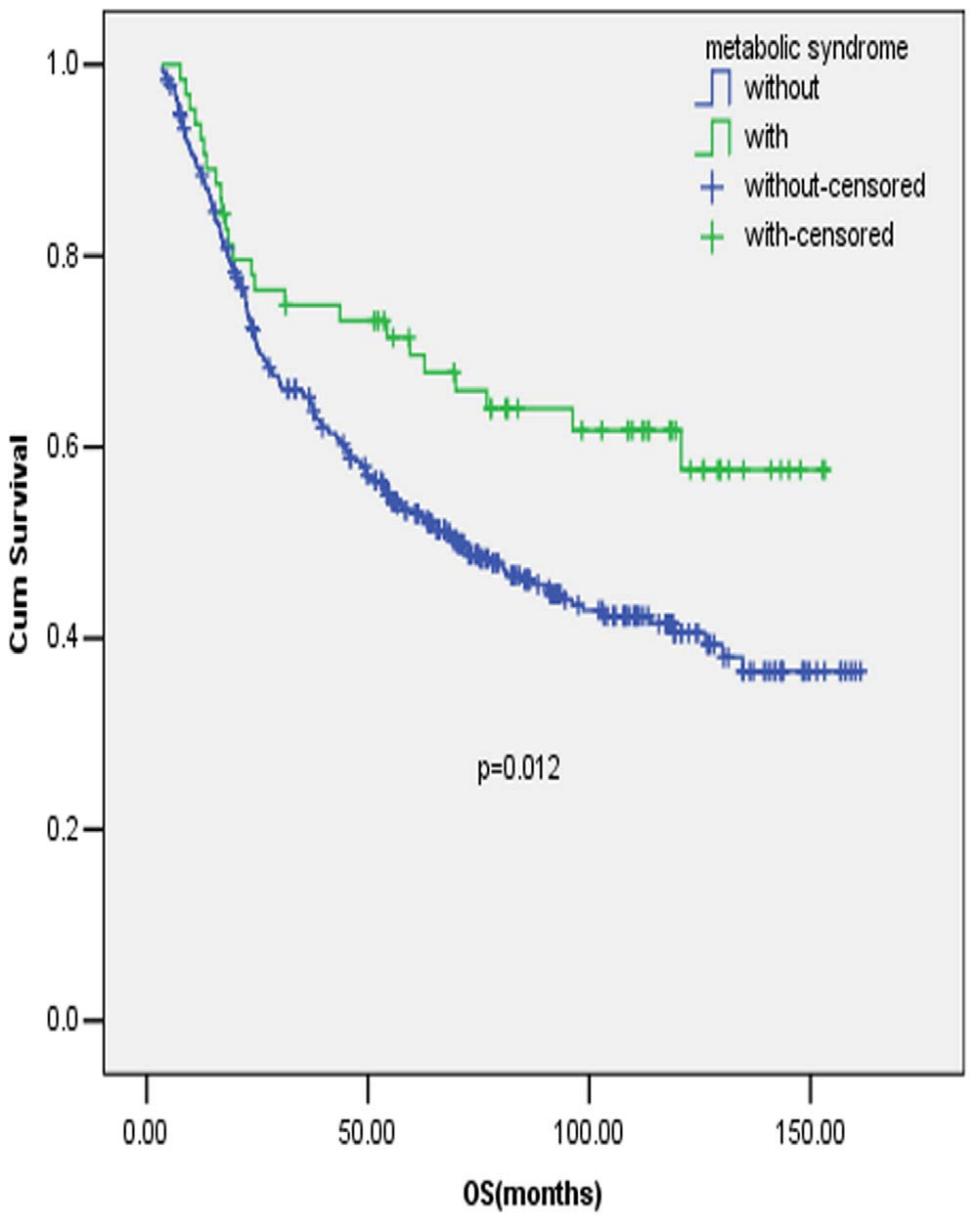
Prognostic value of MetS in early stage gastric cancer patients with old age. Full legend: Survival curves of early stage GC patients with and without MetS in patients aged >50 years old.

**Figure 3:**
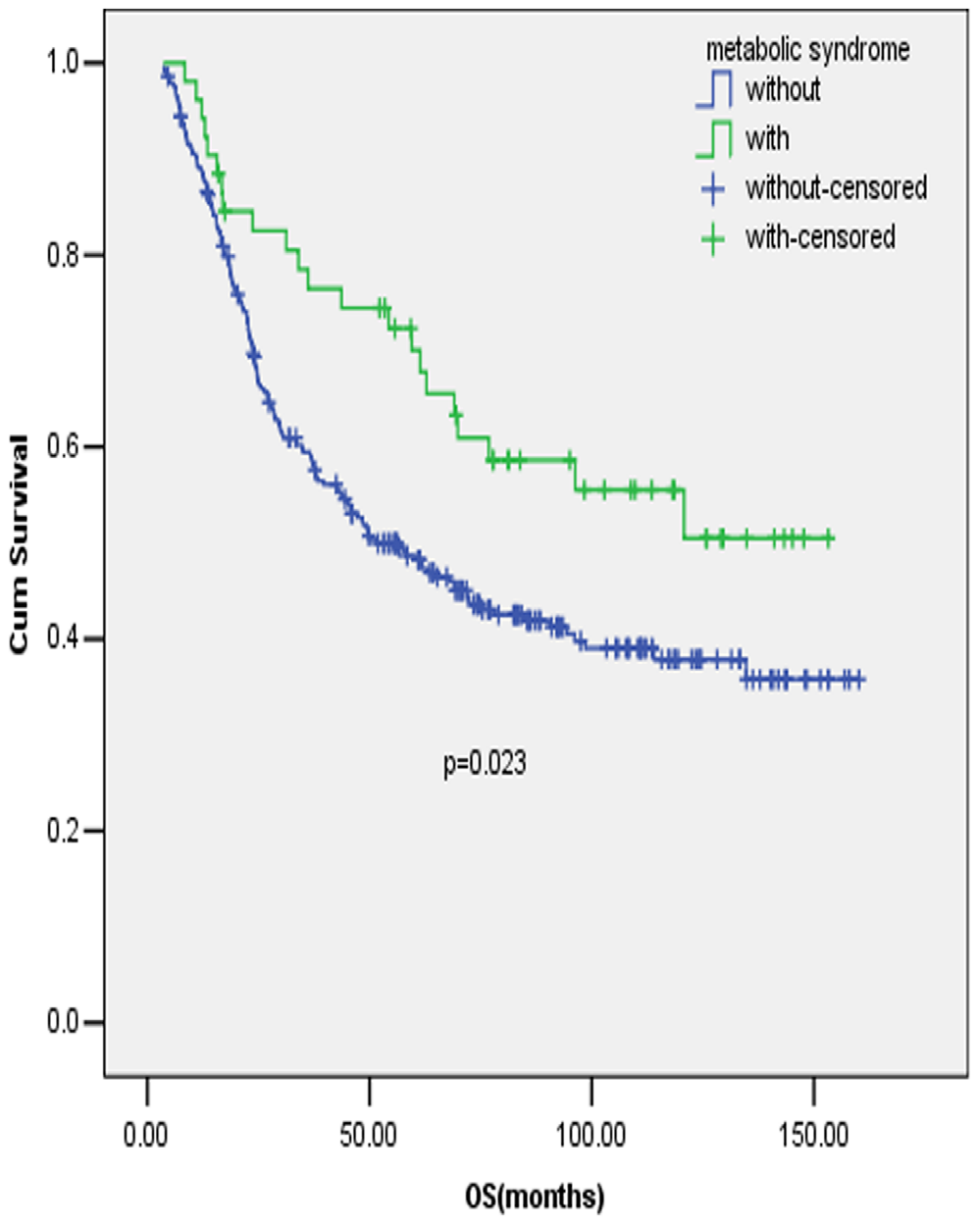
Prognostic value of MetS in early stage gastric cancer patients with proximal tumors. Full legend: Survival curves of early stage GC patients with and without MetS in patients with tumors proximally located.

In patients aged <50 years old, MetS or its components were found to have no significant influence on OS in early stage GC. While in patients aged >50 years old, patients with MetS were found to have a significant better survival compared with patients without MetS (figure 2, P = 0.012), after adjusted by items significant prognostic in the univariate analysis, including tumor location, tumor size, T stage, N stage and surgery type, MetS was identified to be an independent prognostic factor for early stage GC in the multivariate analysis. (Table 2, P = 0.009) Similarly, MetS was found to be a significant and independent prognostic factor in proximal early stage GC (figure 3, P = 0.023 and table 3, P = 0.047), but the association was not identified in distal early stage GC.

**Table 2:** Association between metabolic syndrome (MetS) and its components and overall survival in old patients (age≧50 y) in a univariate and multivariate analysis.

		Univariate analysis		Multivariate analysis	
Factors	Number	Hazard ratio (95% confidence interval)	P value	Hazard ratio (95% confidence interval)	P value
Gender (Male/Female)	320/119	0.786(0.575–1.075)	0.131		
Tumor location(Proximal/Distal)	266/173	0.638(0.480–0.847)	**0.002**	0.634(0.476–0.845)	**0.002**
Tumor size (≤5 cm/>5 cm)	283/156	1.842(1.413–2.401)	**<0.001**	1.528(1.169–1.999)	**0.002**
Degree of differentiation (not differentiated/Poorly differentiated, mucinous or signet ring adenocarcinoma/Moderate differentiated/Well differentiated)	4/311/103/21	0.874(0.694–1.100)	0.250		
T stage(AJCC, 7th)(T1a/T1b/T2/T3/T4a/T4b)	30/7/62/20/273/47	1.019(1.010–1.028)	**<0.001**	1.014(1.006–1.023)	**0.001**
N stage(AJCC, 7th)(N0/N1/N2/N3a/N3b)	162/67/103/78/29	1.037(1.027–1.046)	**<0.001**	1.035(1.025–1.045)	**<0.001**
Surgery type(radical/palliative)	408/31	1.625(1.044–2.529)	**0.031**	1.573(0.998–2.479)	0.051
Angiolymphatic invasion(No/Yes)	413/26	1.388(0.807–2.386)	0.236		
Chemotherapy (No/Yes)	266/173	0.844(0.643–1.107)	0.219		
MetS (No/Yes)	375/64	0.584(0.382–0.893)	**0.012**	0.565(0.368–0.868)	**0.009**
Number of MetS Components			0.166		
0	115	1(Reference)			
1	152	1.007(0.720–1.408)	0.969		
2	108	1.074(0.749–1.538)	0.698		
3	50	0.630(0.380–1.046)	0.074		
4	14	0.476(0.173–1.321)	0.151		
Single component meeting the criteria of MetS					
BMI (No/Yes)	360/79	0.893(0.636–1.253)	0.512		
Fasting plasma glucose or diabetes (No/Yes)	387/52	0.888(0.581–1.357)	0.582		
Blood pressure (No/Yes)	244/195	0.862(0.660–1.125)	0.274		
Triglycerides (No/Yes)	368/71	0.839(0.577–1.221)	0.359		
HDL (No/Yes)	262/177	0.851(0.648–1.118)	0.247		

Abbreviations: AJCC: American Joint Committee on Cancer, MetS: metabolic syndrome, BMI: body mass index, HDL: high density lipoprotein.

**Table 3:** Association between metabolic syndrome (MetS) and overall survival in patients with proximal tumor in a univariate and multivariate analysis.

		Univariate analysis		Multivariate analysis	
Factors	Number	Hazard ratio (95% confidence interval)	P value	Hazard ratio (95% confidence interval)	P value
Gender (Male/Female)	247/91	0.711(0.501–1.010)	0.057		
age(<50/≧50)	72/266	1.371(0.932–2.018)	0.109		
Tumor size (≤5 cm/>5 cm)	204/134	1.785(1.335–2.388)	**<0.001**	1.707(1.272–2.291)	**<0.001**
Degree of differentiation (Poorly differentiated, mucinous or signet ring adenocarcinoma/Moderate differentiated/Well differentiated)	233/88/17	0.781(0.596–1.024)	0.074		
T stage(AJCC, 7th)(T1a/T1b/T2/T3/T4a/T4b)	21/4/46/17/213/37	1.009(1.000–1.018)	**0.041**	1.006(0.997–1.015)	0.222
N stage(AJCC, 7th)(N0/N1/N2/N3a/N3b)	126/52/78/62/20	1.036(1.025–1.046)	**<0.001**	1.035(1.025–1.046)	**<0.001**
Surgery type(radical/palliative)	312/26	1.380(0.857–2.221)	0.186		
Angiolymphatic invasion(No/Yes)	322/16	1.805(0.980–3.323)	0.058		
Chemotherapy (No/Yes)	211/127	0.879(0.651–1.188)	0.402		
MetS (No/Yes)	286/52	0.595(0.381–0.930)	**0.023**	0.635(0.406–0.993)	**0.047**
Number of MetS Components			0.177		
0	93	1(reference)			
1	119	1.034(0.715–1.495)	0.860		
2	74	1.173(0.788–1.748)	0.432		
3	41	0.589(0.340–1.022)	0.060		
4	11	0.758(0.274–2.101)	0.595		
Single component meeting the criteria of MetS					
BMI (No/Yes)	282/56	0.901(0.615–1.320)	0.591		
Fasting plasma glucose or diabetes (No/Yes)	298/40	0.906(0.569–1.442)	0.677		
Blood pressure (No/Yes)	202/136	0.951(0.708–1.278)	0.738		
Triglycerides (No/Yes)	279/59	0.807(0.539–1.207)	0.296		
HDL (No/Yes)	195/143	0.812(0.603–1.093)	0.169		

Abbreviations: AJCC: American Joint Committee on Cancer, MetS: metabolic syndrome, BMI: body mass index, HDL: high density lipoprotein.

**Figure 4:**
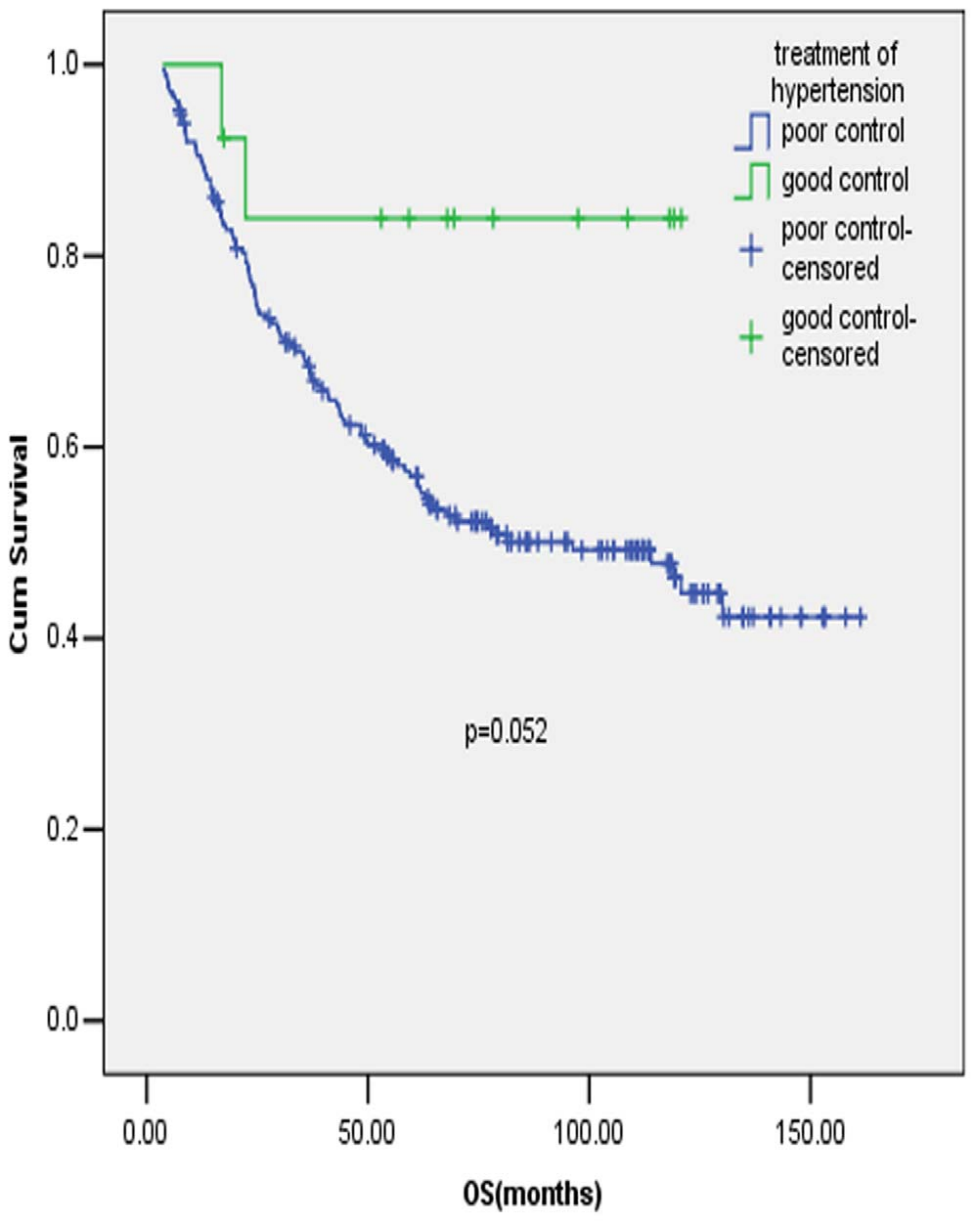
Prognostic value of antihypertension in early stage gastric cancer patients. Full legend: Survival curves of early stage GC patients who had the history of hypertension with blood pressure in good and poor control.

We further explored the effects of the control of MetS disorders on OS in early stage GC. From the medical records, we could only retrospectively find the information about the history of hypertension and diabetes mellitus, thus we couldn't study the effect of treatment for lipid metabolism disturbance on OS. 64 cases were identified to have a history of diabetes mellitus, with 6 (9.38%) cases having the blood glucose controlled to the normal range during the period of GC diagnosis and 58 cases (90.62%) out of control, and whether the blood glucose was controlled or not had no impact on OS in early stage GC. As for the control of blood pressure, among 224 cases with the history of hypertension, 13 (5.80%) cases were classified into the group of good control, and 211 (94.20%) cases in the group of poor control. There was a trend of better survival in the group of good control compared with the group of poor control, but the association was not statistically significant (figure 4, P = 0.052).

## Discussion

Our research found that in early stage (stage I–III) gastric cancer (GC), metabolic syndrome (MetS) was associated with an apparently better differentiation. In addition, MetS predicted a trend of better overall survival (OS) in early stage GC as a whole, but the association was not statistically significant. Further analyses splitting the data by age (<50 y/≧50 y) and tumor location (Proximal/Distal) drew conclusions that in early stage GC, MetS significantly and independently predicted a better survival both in old aged (≧50 y) patients and in patients with tumors proximally located.

There have been a number of studies investigating the association between MetS and/or its components and cancer risks, including GC. It was found that MetS and/or its components were associated with a higher risk of cardiac rather than non-cardiac GC, and the association was not found in early adulthood [21], [22], [33]. The impacts of MetS and/or its components on cancer survivals have been investigated in some other cancers. In prostate and breast cancers, MetS and/or its components were predictors of worse survival [25], [26], [27]. However, analogous studies in another cancer of the digestive system-colon cancer came to controversial conclusions about the importance of MetS in survival. Yang et al. found that elevated glucose or diabetes mellitus and elevated hypertension were predictors of worse survival, in contrast, dyslipidemia was a predictor of better survival, especially in patients with early stage disease in colon cancer [28], [29]. The importance of MetS and/or its components in GC has not been studied so far, hence we focused this study on early stage GC, and the survival analyses were performed in depth through splitting the data by age and tumor location.

Interestingly, our discoveries about the association of MetS and GC were not agreed with some previous conclusions in other cancers. Firstly, we found MetS to be associated with a better differentiation of the GC cells, while some previous studies found MetS and/or its components to be bound up with a more aggressive tumor type in colon and prostate cancer, despite the relevant result was controversial in breast cancer. [25], [34], [35], [36]. Secondly, we identified MetS to be a positive prognostic factor, but a large proportion of studies found MetS negatively predicted the survival in prostate and breast cancers, though the conclusion was inconsistent in colon cancer [25], [27], [28], [29]. Thirdly, when it came to the role of each component, we didn't identify any significant association with OS. In contrast, several previous studies identified elevated blood glucose to be associated with worse prognosis in breast cancer and colon cancer, and elevated blood pressure was associated with worse survival in colon cancer, while dyslipidemia was associated with improved survival in colon cancer [26], [28].

As far as we were concerned, our study was the first to investigate the association between MetS and the malignant grade as well as survival in GC. A previous study by Otani et al. found that lower adipose tissue volume was related to undifferentiated GC rather than differentiated GC [37], it might be a support point for the association of MetS with a better differentiation of the GC cells discovered in our research, owing to MetS was frequently associated with obesity and abnormal lipid metabolism, which indicating a higher adipose tissue volume. In addition, the difference between GC and some other cancers in the relation to the impact of MetS on survival might be attributed to the different tumor properties. Compared with breast, prostate and colon cancers, GC was more aggressive. What's more, GC was located in upper gastrointestinal tract, which meant GC had more influence on patients' nutrition condition. Thus more patients might suffer from malnutrition in GC compared with cancers mentioned above, in turn, a better nutrition status might have more important role to maintain life in GC. More than that, it was found that malnutrition increased the risk of stomach cancer mortality [38]. Compared with patients without MetS, those with MetS were less likely to suffer from Nutrition deficiency. This partly explained why MetS was associated with more favorable OS in GC, which was different from some other cancers. Another explanation for the superior survival in early GC patients with MetS was adiponectin, the level of which was lower in obese compared with lean subjects [39], and obese patients were more frequently associated with MetS. Shin et al. from South Korea found that adiponectin receptor was related to GC development, progression and worse survival [39], although the survival predicting effect was inconsistent with some previous smaller-size studies in Japan and Italy [40], [41]. For the role of each component in GC survival, our finding was in consistent with previous studies about the irrelevance of BMI [30], [32], but concerning about decreased high density lipoprotein, our data didn't show the predictive effect for a worse survival raised by a previous retrospective study with 184 cases [31]. Nevertheless, further investigations are necessary for a better and more accurate understanding of the role of MetS and/or its components on survival in GC.

In the analyses of the influences of the control of MetS disorders on OS in early stage GC, we only studied the associations in hypertension and diabetes mellitus. Due to the limited number of cases included, our conclusions had only limited importance. Although statins have been considered to have anti-tumor effect for a long time, a randomized phase II clinical trial showed no benefit for survival of additional use of pravastatin except for chemotherapy in advanced GC [42]. Despite diabetes was found to be a risk factor for cancer related mortality in GC, insulin use did not affect the outcomes [43], [44]. In addition, as far as we are concerned, there is no evidence for the role of the antihypertensive therapy in GC survival so far. In conclusion, there's plenty of scope to push investigations in this area further.

It was inevitable that there were some limitations in our study. To start with, it was a retrospective research, although all patients enrolled had received radical or palliative operation for GC, we didn't include disease free survival or progress free survival in analysis, because the relevant information might not be credible enough. In addition, the numbers of cases for the analysis of antihypertensive and hypoglycemic therapy were insufficient for dependable conclusions, but we supplied a clue and direction for future researches in this area.

## Conclusions

Metabolic syndrome (MetS) was associated with a more moderate tumor cell differentiation in early stage gastric cancer (GC). Besides, MetS was a significant and independent predictor for better survival in certain groups of early stage GC, including patients with old age (age≧50 y) or patients with proximal GC.
